# Overexpression of BUB1B, CCNA2, CDC20, and CDK1 in tumor tissues predicts poor survival in pancreatic ductal adenocarcinoma

**DOI:** 10.1042/BSR20182306

**Published:** 2019-02-26

**Authors:** Shu Dong, Fei Huang, Hao Zhang, Qiwen Chen

**Affiliations:** 1 Department of Integrative Oncology, Fudan University Shanghai Cancer Center, Shanghai, China; 2 Department of Oncology, Shanghai Medical College, Fudan University, Shanghai 200032, China; 3 Department of Minimally Invasive Oncology, Jiangyin Hepatobiliary Hospital of Traditional Chinese Medicine, Jiangyin 21440, Jiangsu Province, China; 4 Department of Pathology, Fudan University Shanghai Cancer Center, Shanghai, China; 5 Department of Oncology, Shanghai Medical College, Fudan University, Shanghai 200032, China; 6 Institute of Clinical Epidemiology, Key Laboratory of Public Health Safety, Ministry of Education, School of Public Health, Fudan University, Shanghai 200032, China

**Keywords:** cell cycle, disease-free survival, overall survival, pancreatic ductal adenocarcinoma

## Abstract

Overexpressed genes in tumors usually contributed to aggressiveness in pancreatic ductal adenocarcinoma (PDAC). Using Gene Expression Omnibus (GEO) profiles including GSE46234, GSE71989, and GSE107610, we detected overexpressed genes in tumors with R program, which were enriched by Kyoto Encyclopedia of Genes and Genomes (KEGG), Gene ontology (GO), and Reactome pathway databases. Then, we performed a survival analysis of enriched genes based on TCGA profile. Our results revealed that high BUB1B, CCNA2, CDC20, and CDK1 expression in tumors was significantly associated with worse overall survival (OS) (Log rank *P*=0.00338, *P*=0.0447, *P*=0.00965, and *P*=0.00479, respectively), which was validated using a Kaplan–Meier plotter with a median cutoff (Log rank *P*=0.028, *P*=0.0035, *P*=0.039, and *P*=0.0033, respectively). Moreover, overexpression of BUB1B, CCNA2, CDC20, and CDK1 in tumor tissues was significantly associated with disease-free survival (DFS) in PDAC patients (Log rank *P*=0.00565, *P*=0.0357, *P*=0.00104, and *P*=0.00121, respectively). BUB1B, CCNA2, CDC20, and CDK1 were significantly overexpressed in deceased PDAC patients (all *P*<0.01) and in patients with recurrence/disease progression (all *P*<0.05). In addition, PDAC patients with neoplasms of histologic grade G3-4 had significantly higher BUB1B, CCNA2 and CDC20 levels (all *P*<0.05). In conclusion, the up-regulation of BUB1B, CCNA2, CDC20, CDK1, and WEE1 in tumor tissues are associated with worse OS and DFS in PDAC and is correlated with advanced tumor stage and tumor development.

## Introduction

Pancreatic ductal adenocarcinoma (PDAC) arises from the exocrine pancreas and accounts for 95% of all pancreatic cancers [1]. Despite major improvements in its diagnosis and treatment, PDAC remains an aggressive disease that carries a poor prognosis and a 5-year survival rate of approximately 8% in the United States [2,3]. The genetic framework, early metastasis, a dense stroma, propensity for growth in a nutrient-depleted environment, and immunomodulation, all underlie its aggressive nature and resistance to treatment, which makes therapeutic progress a challenge [4]. Hence, it is essential to find predictive biomarkers and novel therapeutic targets to improve the treatment outcome in PDAC patients.

Currently, few tumor markers have been externally validated to predict the survival of patients with PDAC [5]. Novel biomarkers that predict PDAC prognosis and PDAC targets for treatment are urgently required [6]. Recently, big data bioinformatics of molecular targets and networks have gained increased attention [7,8], which is specifically due to the introduction of large-scale molecular analysis platforms [9]. This tremendous amount of molecular data provide a rich source for a better understanding of the molecular basis of PDAC and for the identification of novel genomic targets for therapeutic intervention.

Using the Gene Expression Omnibus (GEO) database, we identified up-regulated differentially expressed genes (DEGs) between tumor tissues and nontumor tissues in PDAC patients, enriched potential pathways/biological processes, and evaluated associations between up-regulated DEGs and PDAC outcomes. We hope our results provide useful insights into potential candidate biomarkers and the pathogenesis and progression of PDAC.

## Materials and methods

### Study design and source of data

**Figure 1:**
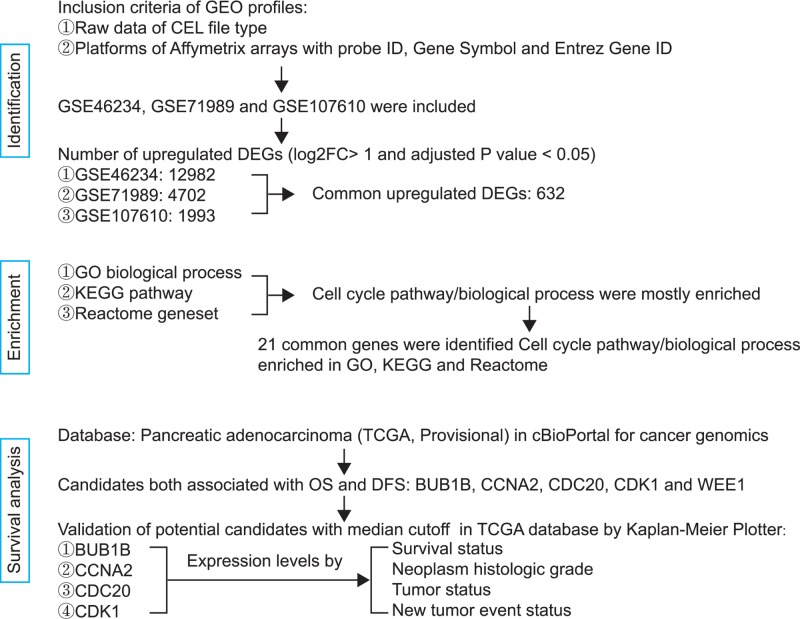
Flow diagram of the analysis procedure

The flowchart of the procedure is described in Figure 1. GEO (https://www.ncbi.nlm.nih.gov/geo/) profiles with raw data of the CEL file type and platforms of Affymetrix arrays with probe ID, Gene Symbol, and Entrez Gene ID were included in this analysis. The gene expression profiles of GSE46234, GSE71989 and GSE107610 were downloaded from the GEO database. The GSE46234 profile was composed of four healthy tissues and four PDAC tissues. GSE71989 included 8 normal pancreatic tissues and 14 PDAC tissues. Data from GSE71989 were generated from Affymetrix arrays. In GSE107610, mRNA from 39 patient-derived PDAC tumors and 2 normal organs was extracted [10] and hybridized to the GeneChip PrimeView Human Gene Expression Array and were scanned using a GeneChip Scanner 3000 7G.

### Identification of up-regulated DEGs in PDAC

To investigate DEGs between tumor tissues and nontumor tissues in PDAC patients, the transcriptome gene expression data using the robust multiarray average (RMA) algorithm were explored. Bioconductor (http://www.bioconductor.org) packages Affy and AffyPLM [11,12] were used for quality assessment of tumor and nontumor samples in each GEO profile. The Limma package [13,14] in Bioconductor was used to identify DEGs (∣log_2_FC∣ > 1, adjusted *P*-value <0.05). To identify up-regulated DEGs, log_2_FC > 1 and adjusted *P*-values <0.05 were set for each GEO profile. To identify shared DEGs amongst GSE46234, GSE71989, and GSE107610, the Venny 2.1 online service (http://bioinfogp.cnb.csic.es/tools/venny/index.html) was used to generate a Venn diagram.

### Functional enrichment analysis

Kyoto Encyclopedia of Genes and Genomes (KEGG), Gene ontology (GO), and Reactome enrichment analysis of up-regulated DEGs was conducted using Gene Set Enrichment Analysis (GSEA). To investigate gene sets, up-regulated DEGs were uploaded to the Molecular Signatures Database in GSEA. A false discovery rate *P*-value cutoff of <0.05 was set as the screening condition. The shared up-regulated DEGs in common pathways enriched by KEGG, GO, and Reactome were determined for the Venn diagram using the Venny 2.1 online service (http://bioinfogp.cnb.csic.es/tools/venny/index.html) [15].

### Survival analysis

To identify potential candidate biomarkers for overall survival (OS) and disease-free survival (DFS) in PDAC patients, the pancreatic adenocarcinoma (TCGA, Provisional) database in cBioPortal for Cancer Genomics web interface was used [16,17]. A z-score threshold ±2.0 of mRNA expression was selected in genomic profiles, and 178 cases with sequenced tumors were included in the survival analysis. The mRNA expression levels of potential candidate biomarkers that were calculated by log_2_ were compared based on clinical factors in PDAC patients. For validation of prognostic candidates in PDAC survival, a Kaplan–Meier analysis (http://kmplot.com/analysis/) with a median cutoff was used [18,19].

### Statistical analysis

Differences of gene expression between the individual groups were analyzed using Mann–Whitney U test and Student’s *t* test based on variables’ types. GraphPad Prism version 7.0 (GraphPad Software, San Diego, CA) was used. A two-tailed *P*<0.05 were considered significant for all tests.

## Results

### Screening up-regulated expressed genes at the mRNA level

**Figure 2:**
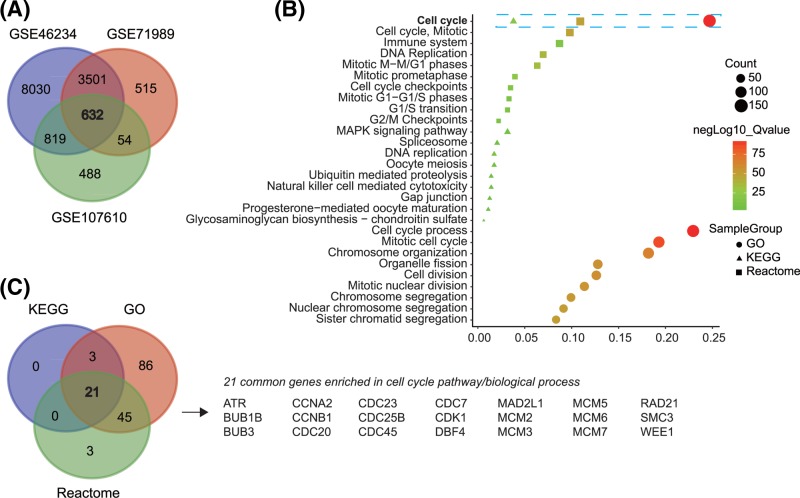
Common up-regulation genes identification and gene functional enrichment Identification of common up-regulated genes in GSE46234, GSE71989, and GSE107610 (**A**), function enrichment of common up-regulated genes (**B**), and Venny diagram of genes enriched in cell cycle pathway/biological process (**C**).

The quality assessments of GSE46234, GSE71989, and GSE107610 were conducted by Affy and AffyPLM packages using relative log expression (RLE) and normalized unscaled standard errors (NUSE). As shown in Supplementary Figures S1–S3, the quality of GSE46234, GSE71989, and GSE107610 was reliable. Amongst GSE46234, GSE71989, and GSE107610, 632 common up-regulated DEGs were identified using a Venn diagram (Figure 2A).

### Up-regulated gene functions and pathways

The KEGG pathway, GO biological process, and Reactome gene sets were analyzed for enrichment of up-regulated gene functions and pathways [20,21]. We presented the top ten pathways/biological processes in our study. The cell cycle was the most enriched pathway/biological process in KEGG, GO, and Reactome (Figure 2B). Additionally, 24 genes were enriched in KEGG pathways, 155 genes were enriched in GO biological processes, and 69 were enriched in Reactome gene sets. Subsequently, we generated a Venn diagram and found that 21 genes including *ART, BUB1B, BUB3, CCNA2, CCNB1, CDC20, CDC23, CDC25B, CDC45, CDC7, CDK1, DBF4, MAD2L1, MCM2, MCM3, MCM5, MCM6, MCM7, RAD21, SMC3*, and *WEE1* in the cell cycle pathway were shared in the three enrichment databases (Figure 2C).

### Up-regulated BUB1B, CCNA2, CDC20, and CDK1 predict worse survival in PDAC

**Figure 3:**
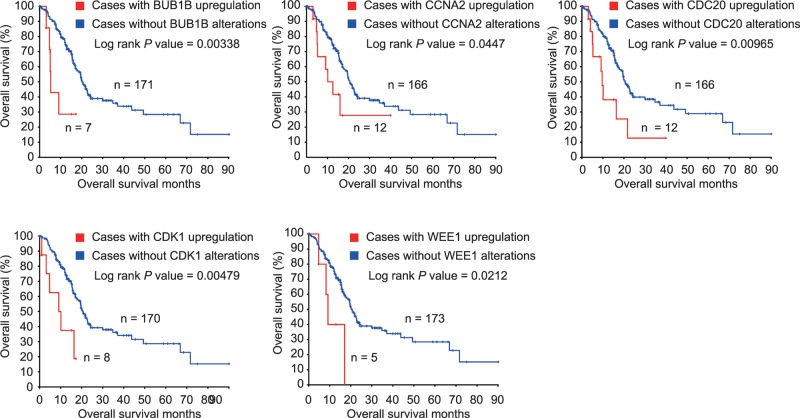
OS of PDAC patients grouped by BUB1B, CCNA2, CDC20, CDK1, and WEE1 in cBioPortal

**Figure 4:**
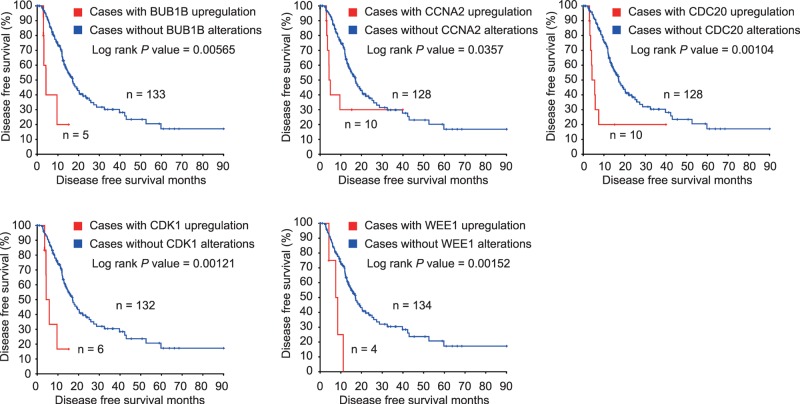
DFS of PDAC patients grouped by BUB1B, CCNA2, CDC20, CDK1, and WEE1 in cBioPortal

Using the pancreatic adenocarcinoma (TCGA, Provisional) database in cBioPortal for Cancer Genomics web interface, we included the 21 enriched genes mentioned above to identify potential candidate biomarkers for OS and DFS in PDAC patients. Only genes that were significantly associated with both OS and DFS were considered potential biomarkers for PDAC prognosis. PDAC patients with high BUB1B, CCNA2, CDC20, CDK1, and WEE1 in tumors experienced worse OS (Log rank *P*=0.00338, *P*=0.0447, *P*=0.00965, *P*=0.00479, and *P=*0.0212, respectively, Figure 3). Similarly, overexpression of BUB1B, CCNA2, CDC20, CDK1, and WEE1 in tumors was significantly associated with DFS in PDAC patients (Log rank *P*=0.00565, *P*=0.0357, *P=*0.00104, *P=*0.00121, and *P*=0.00152, for BUB1B, CCNA2, CDC20, CDK1, and WEE1, respectively, Figure 4).

**Figure 5:**
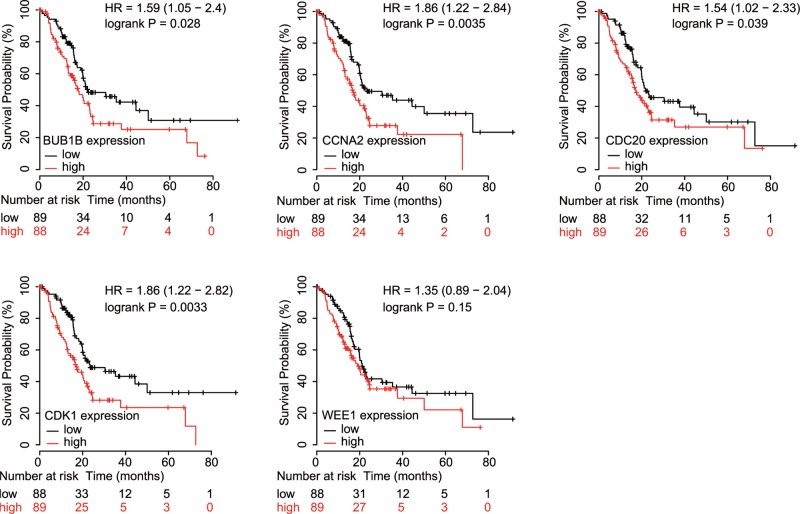
OS validation of PDAC patients grouped by median cutoffs of BUB1B, CCNA2, CDC20, CDK1, and WEE1 in Kaplan–Meier plotter

For validation, we conducted a subgroup analysis using median cutoffs of BUB1B, CCNA2, CDC20, CDK1, and WEE1 in a Kaplan–Meier plot. As shown in Figure 5, overexpression of BUB1B, CCNA2, CDC20, and CDK1 in tumors was significantly associated with worse OS in PDAC patients (HR = 1.59, 95% CI = 1.05–2.4, *P*=0.028; HR = 1.86, 95% CI = 1.22–2.84, *P*=0.0035; HR = 1.54, 95% CI = 1.02–2.33, *P*=0.039; HR = 1.86, 95% CI = 1.22–2.82, *P*=0.0033; respectively, Figure 5). Unfortunately, no significance was found between WEE1 expression and OS in PDAC patients (HR = 1.35, 95% CI = 0.89–2.04, *P*=0.15, Figure 5).

**Figure 6:**
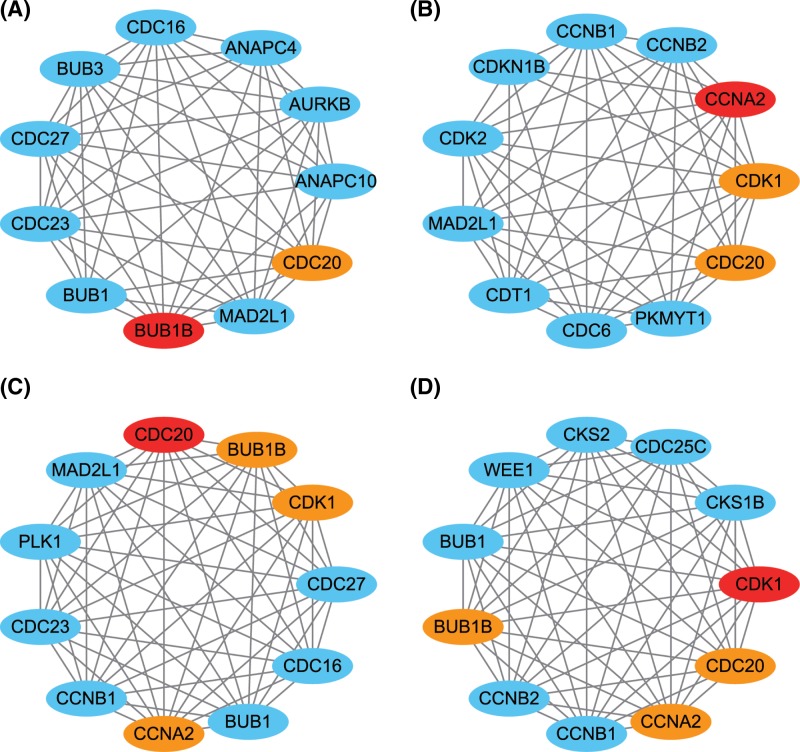
Protein-protein interaction analysis Protein–protein interaction network of BUB1B (**A**), CCNA2 (**B**), CDC20 (**C**), and CDK1 (**D**).

Considering the results above, we cautiously concluded that up-regulated BUB1B, CCNA2, CDC20, and CDK1 in tumors predict worse survival of PDAC patients. In addition, we performed a protein–protein interaction network analysis of BUB1B, CCNA2, CDC20, and CDK1. As shown in Figure 6, these four genes mostly interact with cell cycle genes and should serve as a panel for the development of malignancies.

### Associations between BUB1B, CCNA2, CDC20, and CDK1, and clinicopathological characteristics of PDAC

**Figure 7:**
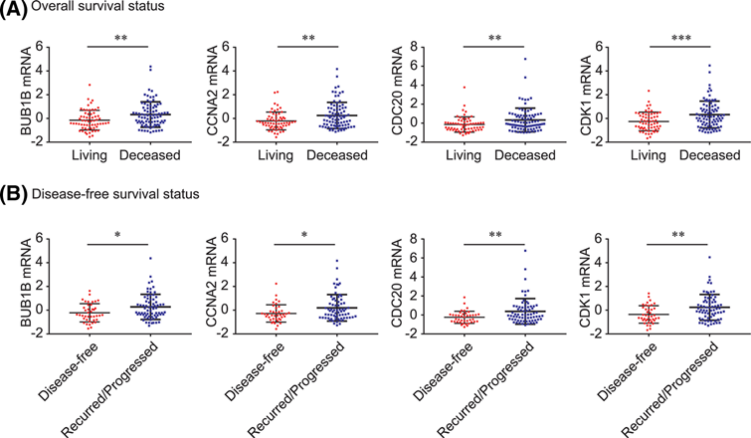
BUB1B, CCNA2, CDC20, CDK1 and WEE1 expression by survival status BUB1B, CCNA2, CDC20, CDK1 and WEE1 comparison by OS status (**A**) and DFS status (**B**). ^*^P<0.05; ^**^P<0.01;^***^P<0.001.

**Figure 8:**
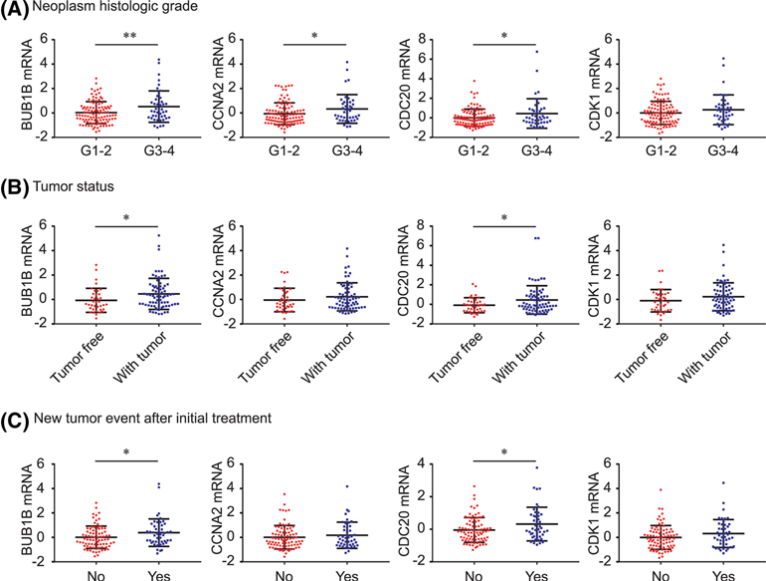
BUB1B, CCNA2, CDC20, CDK1 and WEE1 expression by clinico-pathological features BUB1B, CCNA2, CDC20, CDK1, and WEE1 comparison by neoplasm histologic grade (**A**), tumor status (**B**), and new tumor event status (**C**). ^*^P<0.05; ^**^P<0.01.

As shown in Figure 7, BUB1B, CCNA2, CDC20, and CDK1 were significantly overexpressed in deceased PDAC patients (all *P*<0.01, Figure 7A) and in patients who experienced recurrence/progression (all *P*<0.05, Figure 7B). In addition, PDAC patients with neoplasms of histologic grade G3-4 had significantly higher BUB1B, CCNA2, and CDC20 levels than those with grade G1-2 neoplasms (all *P*<0.05, Figure 8A), and high levels of BUB1B and CDC20 contributed to tumor formation (both *P*<0.05, Figure 8B). Similarly, BUB1B and CDC20 were both significantly up-regulated in PDAC patients with new tumor development after initial treatment (both *P*<0.05, Figure 8C).

## Discussion

PDAC is amongst the most important unresolved health problems worldwide and is a lethal disease partly due to a lack of therapeutic treatment targets [22]. To identify prognostic factors that can stratify patients according to biological markers may help in the discovery of novel therapeutic approaches and the selection of adequate treatment strategies [23,24]. Unfortunately, amongst the current prognostic factors, few have been translated into clinical practice [5].

Consistent with previous reports [25,26], we found that when advanced tumor biological behaviors, including the histologic grade of the neoplasm and tumor development, are considered, BUB1B, CCNA2, CDC20, and CDK1 were enriched in the cell cycle biological process/pathway and were associated with OS and DFS in PDAC patients. This indicates that, based on the data shown in the present study, BUB1B, CCNA2, CDC20, and CDK1 show prognostic value for PDAC patients.

Encoded by BUB1B, BUBR1 expression is sufficient to predict poor prognosis in pancreatobiliary-type tumors [27]. Previous bioinformatics analyses showed that BUB1B was one of the hub genes with high degrees of connectivity to PDAC and might be a potential target for PDAC diagnosis and treatment [25]. However, the role of BUB1B in other types of cancer cells is still controversial. Low expression of BUB1B contributes to poor survival and metastasis in human colon adenocarcinomas [28] and several lung cancer cell lines [29], while overexpression of BUB1B is related to progression and recurrence of gastric cancer [30], bladder cancer [31], liver cancer [32], and many other cancers [33–35]. CCNA2 belongs to a highly conserved cyclin family and is up-regulated in dozens of cancer types, which indicates its potential roles in cancer transformation and progression [36]. It has been reported that a high CCNA2 expression promotes cell proliferation in hepatoma [37] and might help monitor chemotherapy efficacy in breast cancer [38]. A bioinformatics analysis by Zhou et al. [26] revealed that CCNA2 overexpression was tightly related to progression of PDAC. Considering the above findings, we believe that BUB1B and CCNA2 should be novel prognostic biomarkers in PDAC.

Overexpression of CDC20 has been reported in various malignancies and high expression of CDC20 has been associated with high tumor grade in bladder, cervical, colon, endometrial, gastric, liver, ovarian, prostatic, and renal carcinomas [39]. CDC20 has been reported to be significantly associated with poor prognosis in pancreatic [40], lung [41], bladder [42], colon [43], oral squamous cell carcinomas [44], and breast cancers [45]. In one study, overexpression of CDC20 enhanced cell proliferation and invasion, while down-regulation of CDC20 promoted anti-tumor activity in pancreatic cancer cells [46]. Hence, CDC20 may represent a promising therapeutic target in cancer patients including those with PDAC [47]. CDK1 has been reported to be correlated with cancer growth and is a key cell cycle regulator [48]. CDK1 expression and activity are elevated in colorectal cancer [49], prostate cancer [50], and lymphomas [51,52]. Up-regulation of CDK1 is associated with poor prognosis in breast cancer [53] and epithelial ovarian cancer [54,55]. In one study, CDK1 inhibitors contributed to a marked reduction in the proportion of cells in S and G_2_/M phases of the cell cycle in PDAC tumor cell models; targetting CDK1 also showed promising anticancer activity in pancreatic cancer cells [56,57].

## Conclusion

Our preliminary analysis showed that BUB1B, CCNA2, CDC20, and CDK1 were overexpressed in tumors in PDAC patients. And these four genes were associated with advanced tumor stage and showed prognostic values for PDAC outcomes. Based on our results, we cautiously concluded that BUB1B, CCNA2, CDC20, and CDK1 should comprise a panel for PDAC development and that they might be novel treatment targets. Since the sample size in this preliminary analysis was small, prospective studies with large samples should be considered for the validation of our results.

## Supporting information

**Figure S1:** Relative log expression (A) and Normalized unscaled standard errors (B) assessment of GSE46234

**Figure S2:** Relative log expression (A) and Normalized unscaled standard errors (B) assessment of GSE71989

**Figure S3:** Relative log expression (A) and Normalized unscaled standard errors (B) assessment of GSE107610

## Abbreviations

BUB1B: BUB1 mitotic checkpoint serine/threonine kinase B
CCNA2: Cyclin A2
CDC20: Cell division cycle 20
CDK1: Cyclin dependent kinase 1
CI: Confidence interval
DEG: Differentially expressed gene
DFS: Disease-free survival
GEO: Gene Expression Omnibus
GO: Gene ontology
GSEA: Gene Set Enrichment Analysis
KEGG: Kyoto Encyclopedia of Genes and Genomes
OS: Overall survival
PDAC: Pancreatic ductal adenocarcinoma
TCGA: The Cancer Genome Atlas
WEE1: WEE1 G2 checkpoint kinase

## References

[1] Becker A.E., Hernandez Y.G., Frucht H. and Lucas A.L. (2014) Pancreatic ductal adenocarcinoma: risk factors, screening, and early detection. World J. Gastroenterol. 20, 11182–11198 10.3748/wjg.v20.i32.11182 25170203 PMC4145757

[2] Siegel R.L., Miller K.D. and Jemal A. (2017) Cancer Statistics, 2017. CA Cancer J. Clin. 67, 7–30 10.3322/caac.21387 28055103

[3] Bray F., Ferlay J., Soerjomataram I., Siegel R.L., Torre L.A. and Jemal A. (2018) Global Cancer Statistics 2018: GLOBOCAN Estimates of Incidence and Mortality Worldwide for 36 Cancers in 185 Countries. CA Cancer J. Clin., 68, 394–424 10.3322/caac.21492 30207593

[4] Ryan D.P., Hong T.S. and Bardeesy N. (2014) Pancreatic adenocarcinoma. N. Engl. J. Med. 371, 1039–1049 10.1056/NEJMra1404198 25207767

[5] Le N., Sund M., Vinci A. and GEMS collaborating group of Pancreas 2000 (2016) Prognostic and predictive markers in pancreatic adenocarcinoma. Dig. Liver Dis. 48, 223–230 10.1016/j.dld.2015.11.001 26769569

[6] Russo S.M., Ove R. and Saif M.W. (2011) Identification of prognostic and predictive markers in pancreatic adenocarcinoma. Highlights from the “2011 ASCO Gastrointestinal Cancers Symposium”. San Francisco, CA, USA. January 20-22, 2011. JOP 12, 92–95 21386628

[7] Andreu-Perez J., Poon C.C., Merrifield R.D., Wong S.T. and Yang G.Z. (2015) Big data for health. IEEE J. Biomed. Health Inform. 19, 1193–1208 10.1109/JBHI.2015.2450362 26173222

[8] Greene C.S., Tan J., Ung M., Moore J.H. and Cheng C. (2014) Big data bioinformatics. J. Cell. Physiol. 229, 1896–1900 10.1002/jcp.24662 24799088 PMC5604462

[9] Merrick B.A., London R.E., Bushel P.R., Grissom S.F. and Paules R.S. (2011) Platforms for biomarker analysis using high-throughput approaches in genomics, transcriptomics, proteomics, metabolomics, and bioinformatics. IARC Sci. Publish. 163, 121–142 22997859

[10] Seino T., Kawasaki S., Shimokawa M., Tamagawa H., Toshimitsu K., Fujii M. (2018) Human pancreatic tumor organoids reveal loss of stem cell niche factor dependence during disease progression. Cell Stem Cell 22, 454–467.e456, 10.1016/j.stem.2017.12.009 29337182

[11] Gautier L., Cope L., Bolstad B.M. and Irizarry R.A. (2004) affy–analysis of Affymetrix GeneChip data at the probe level. Bioinformatics 20, 307–315 10.1093/bioinformatics/btg405 14960456

[12] Gentleman R.C., Carey V.J., Bates D.M., Bolstad B., Dettling M., Dudoit S. (2004) Bioconductor: open software development for computational biology and bioinformatics. Genome Biol. 5, R80 10.1186/gb-2004-5-10-r80 15461798 PMC545600

[13] Ritchie M.E., Phipson B., Wu D., Hu Y., Law C.W., Shi W. (2015) limma powers differential expression analyses for RNA-sequencing and microarray studies. Nucleic Acids Res. 43, e47 10.1093/nar/gkv007 25605792 PMC4402510

[14] Diboun I., Wernisch L., Orengo C.A. and Koltzenburg M. (2006) Microarray analysis after RNA amplification can detect pronounced differences in gene expression using limma. BMC Genomics 7, 252 10.1186/1471-2164-7-252 17029630 PMC1618401

[15] Oliveros J.C. Venny. An interactive tool for comparing lists with Venn’s diagrams, (2007) http://bioinfogp.cnb.csic.es/tools/venny/index.html

[16] Gao J., Aksoy B.A., Dogrusoz U., Dresdner G., Gross B., Sumer S.O. (2013) Integrative analysis of complex cancer genomics and clinical profiles using the cBioPortal. Sci. Signal. 6, pl1 10.1126/scisignal.2004088 23550210 PMC4160307

[17] Cerami E., Gao J., Dogrusoz U., Gross B.E., Sumer S.O., Aksoy B.A. (2012) The cBio cancer genomics portal: an open platform for exploring multidimensional cancer genomics data. Cancer Discov. 2, 401–404 10.1158/2159-8290.CD-12-0095 22588877 PMC3956037

[18] Nagy A., Lanczky A., Menyhart O. and Gyorffy B. (2018) Validation of miRNA prognostic power in hepatocellular carcinoma using expression data of independent datasets. Sci. Rep. 8, 9227 10.1038/s41598-018-27521-y 29907753 PMC6003936

[19] Szasz A.M., Lanczky A., Nagy A., Forster S., Hark K., Green J.E. (2016) Cross-validation of survival associated biomarkers in gastric cancer using transcriptomic data of 1,065 patients. Oncotarget 7, 49322–49333 10.18632/oncotarget.10337 27384994 PMC5226511

[20] Mootha V.K., Lindgren C.M., Eriksson K.F., Subramanian A., Sihag S., Lehar J. (2003) PGC-1alpha-responsive genes involved in oxidative phosphorylation are coordinately downregulated in human diabetes. Nat. Genet. 34, 267–273 10.1038/ng1180 12808457

[21] Subramanian A., Tamayo P., Mootha V.K., Mukherjee S., Ebert B.L., Gillette M.A. (2005) Gene set enrichment analysis: a knowledge-based approach for interpreting genome-wide expression profiles. Proc. Natl. Acad. Sci. U.S.A. 102, 15545–15550 10.1073/pnas.0506580102 16199517 PMC1239896

[22] Seufferlein T., Bachet J.B., Van Cutsem E., Rougier P. and Group E.G.W. (2012) Pancreatic adenocarcinoma: ESMO-ESDO Clinical Practice Guidelines for diagnosis, treatment and follow-up. Ann. Oncol. 23, vii33–vii40 10.1093/annonc/mds224 22997452

[23] Dunne R.F. and Hezel A.F. (2015) Genetics and biology of pancreatic ductal adenocarcinoma. Hematol. Oncol. Clin. North Am. 29, 595–608 10.1016/j.hoc.2015.04.003 26226899 PMC5697145

[24] Ying H., Dey P., Yao W., Kimmelman A.C., Draetta G.F., Maitra A. (2016) Genetics and biology of pancreatic ductal adenocarcinoma. Genes Dev. 30, 355–385 10.1101/gad.275776.115 26883357 PMC4762423

[25] Long J., Zhang Z., Liu Z., Xu Y. and Ge C. (2016) Identification of genes and pathways associated with pancreatic ductal adenocarcinoma by bioinformatics analyses. Oncol. Lett. 11, 1391–1397 10.3892/ol.2015.4042 26893748 PMC4734321

[26] Zhou Z., Cheng Y., Jiang Y., Liu S., Zhang M., Liu J. (2018) Ten hub genes associated with progression and prognosis of pancreatic carcinoma identified by co-expression analysis. Int. J. Biol. Sci. 14, 124–136 10.7150/ijbs.22619 29483831 PMC5821034

[27] Gladhaug I.P., Westgaard A., Schjolberg A.R., Burum-Auensen E., Pomianowska E. and Clausen O.P. (2010) Spindle proteins in resected pancreatic head adenocarcinomas: BubR1 is an independent prognostic factor in pancreatobiliary-type tumours. Histopathology 56, 345–355 10.1111/j.1365-2559.2010.03489.x 20459534

[28] Shichiri M., Yoshinaga K., Hisatomi H., Sugihara K. and Hirata Y. (2002) Genetic and epigenetic inactivation of mitotic checkpoint genes hBUB1 and hBUBR1 and their relationship to survival. Cancer Res. 62, 13–17 11782350

[29] Park H.Y., Jeon Y.K., Shin H.J., Kim I.J., Kang H.C., Jeong S.J. (2007) Differential promoter methylation may be a key molecular mechanism in regulating BubR1 expression in cancer cells. Exp. Mol. Med. 39, 195–204 10.1038/emm.2007.22 17464181

[30] Ando K., Kakeji Y., Kitao H., Iimori M., Zhao Y., Yoshida R. (2010) High expression of BUBR1 is one of the factors for inducing DNA aneuploidy and progression in gastric cancer. Cancer Sci. 101, 639–645 10.1111/j.1349-7006.2009.01457.x 20132214 PMC11159402

[31] Yamamoto Y., Matsuyama H., Chochi Y., Okuda M., Kawauchi S., Inoue R. (2007) Overexpression of BUBR1 is associated with chromosomal instability in bladder cancer. Cancer Genet. Cytogenet. 174, 42–47 10.1016/j.cancergencyto.2006.11.012 17350465

[32] Liu A.W., Cai J., Zhao X.L., Xu A.M., Fu H.Q., Nian H. (2009) The clinicopathological significance of BUBR1 overexpression in hepatocellular carcinoma. J. Clin. Pathol. 62, 1003–1008 10.1136/jcp.2009.066944 19861558

[33] Fu X., Chen G., Cai Z.D., Wang C., Liu Z.Z., Lin Z.Y. (2016) Overexpression of BUB1B contributes to progression of prostate cancer and predicts poor outcome in patients with prostate cancer. Onco. Targets Ther. 9, 2211–2220 27143916 10.2147/OTT.S101994 PMC4844448

[34] Tanaka K., Mohri Y., Ohi M., Yokoe T., Koike Y., Morimoto Y. (2008) Mitotic checkpoint genes, hsMAD2 and BubR1, in oesophageal squamous cancer cells and their association with 5-fluorouracil and cisplatin-based radiochemotherapy. Clin. Oncol. (R. Coll. Radiol.) 20, 639–646 10.1016/j.clon.2008.06.010 18691855

[35] Yuan B., Xu Y., Woo J.H., Wang Y., Bae Y.K., Yoon D.S. (2006) Increased expression of mitotic checkpoint genes in breast cancer cells with chromosomal instability. Clin. Cancer Res. 12, 405–410 10.1158/1078-0432.CCR-05-0903 16428479

[36] Uhlen M., Oksvold P., Fagerberg L., Lundberg E., Jonasson K., Forsberg M. (2010) Towards a knowledge-based human protein atlas. Nat. Biotechnol. 28, 1248–1250 10.1038/nbt1210-1248 21139605

[37] Yang F., Gong J., Wang G., Chen P., Yang L. and Wang Z. (2016) Waltonitone inhibits proliferation of hepatoma cells and tumorigenesis via FXR-miR-22-CCNA2 signaling pathway. Oncotarget 7, 75165–75175 10.18632/oncotarget.12614 27738335 PMC5342731

[38] Gao T., Han Y., Yu L., Ao S., Li Z. and Ji J. (2014) CCNA2 is a prognostic biomarker for ER+ breast cancer and tamoxifen resistance. PLoS ONE 9, e91771 10.1371/journal.pone.0091771 24622579 PMC3951414

[39] Gayyed M.F., El-Maqsoud N.M., Tawfiek E.R., El Gelany S.A. and Rahman M.F. (2016) A comprehensive analysis of CDC20 overexpression in common malignant tumors from multiple organs: its correlation with tumor grade and stage. Tumour Biol. 37, 749–762 10.1007/s13277-015-3808-1 26245990

[40] Chang D.Z., Ma Y., Ji B., Liu Y., Hwu P., Abbruzzese J.L. (2012) Increased CDC20 expression is associated with pancreatic ductal adenocarcinoma differentiation and progression. J. Hematol. Oncol. 5, 15 10.1186/1756-8722-5-15 22475564 PMC3350393

[41] Kato T., Daigo Y., Aragaki M., Ishikawa K., Sato M. and Kaji M. (2012) Overexpression of CDC20 predicts poor prognosis in primary non-small cell lung cancer patients. J. Surg. Oncol. 106, 423–430 10.1002/jso.23109 22488197

[42] Choi J.W., Kim Y., Lee J.H. and Kim Y.S. (2013) High expression of spindle assembly checkpoint proteins CDC20 and MAD2 is associated with poor prognosis in urothelial bladder cancer. Virchows Arch. 463, 681–687 10.1007/s00428-013-1473-6 23995871

[43] Wu W.J., Hu K.S., Wang D.S., Zeng Z.L., Zhang D.S., Chen D.L. (2013) CDC20 overexpression predicts a poor prognosis for patients with colorectal cancer. J. Transl. Med. 11, 142 10.1186/1479-5876-11-142 23758705 PMC3691738

[44] Moura I.M., Delgado M.L., Silva P.M., Lopes C.A., do Amaral J.B., Monteiro L.S. (2014) High CDC20 expression is associated with poor prognosis in oral squamous cell carcinoma. J. Oral Pathol. Med. 43, 225–231 10.1111/jop.12115 24044615

[45] Karra H., Repo H., Ahonen I., Loyttyniemi E., Pitkanen R., Lintunen M. (2014) Cdc20 and securin overexpression predict short-term breast cancer survival. Br. J. Cancer 110, 2905–2913 10.1038/bjc.2014.252 24853182 PMC4056061

[46] Zhang Y., Xue Y.B., Li H., Qiu D., Wang Z.W. and Tan S.S. (2017) Inhibition of cell survival by curcumin is associated with downregulation of cell division cycle 20 (Cdc20) in pancreatic cancer cells. Nutrients 9, E109 10.3390/nu9020109 28165402 PMC5331540

[47] Wang Z., Wan L., Zhong J., Inuzuka H., Liu P., Sarkar F.H. (2013) Cdc20: a potential novel therapeutic target for cancer treatment. Curr. Pharm. Des. 19, 3210–3214 10.2174/1381612811319180005 23151139 PMC4014638

[48] Santamaria D., Barriere C., Cerqueira A., Hunt S., Tardy C., Newton K. (2007) Cdk1 is sufficient to drive the mammalian cell cycle. Nature 448, 811–815 10.1038/nature06046 17700700

[49] Sung W.W., Lin Y.M., Wu P.R., Yen H.H., Lai H.W., Su T.C. (2014) High nuclear/cytoplasmic ratio of Cdk1 expression predicts poor prognosis in colorectal cancer patients. BMC Cancer 14, 951 10.1186/1471-2407-14-951 25511643 PMC4302138

[50] Willder J.M., Heng S.J., McCall P., Adams C.E., Tannahill C., Fyffe G. (2013) Androgen receptor phosphorylation at serine 515 by Cdk1 predicts biochemical relapse in prostate cancer patients. Br. J. Cancer 108, 139–148 10.1038/bjc.2012.480 23321516 PMC3553508

[51] Tsaur I., Makarevic J., Hudak L., Juengel E., Kurosch M., Wiesner C. (2011) The cdk1-cyclin B complex is involved in everolimus triggered resistance in the PC3 prostate cancer cell line. Cancer Lett. 313, 84–90 10.1016/j.canlet.2011.08.026 21925791

[52] Banerjee S.K., Weston A.P., Zoubine M.N., Campbell D.R. and Cherian R. (2000) Expression of cdc2 and cyclin B1 in *Helicobacter pylori*-associated gastric MALT and MALT lymphoma: relationship to cell death, proliferation, and transformation. Am. J. Pathol. 156, 217–225 10.1016/S0002-9440(10)64722-0 10623670 PMC1868611

[53] Li Y., Chen Y.L., Xie Y.T., Zheng L.Y., Han J.Y., Wang H. (2013) Association study of germline variants in CCNB1 and CDK1 with breast cancer susceptibility, progression, and survival among Chinese Han women. PLoS ONE 8, e84489 10.1371/journal.pone.0084489 24386390 PMC3873991

[54] Yang W., Cho H., Shin H.Y., Chung J.Y., Kang E.S., Lee E.J. (2016) Accumulation of cytoplasmic Cdk1 is associated with cancer growth and survival rate in epithelial ovarian cancer. Oncotarget 7, 49481–49497 27385216 10.18632/oncotarget.10373 PMC5226523

[55] Xi Q., Huang M., Wang Y., Zhong J., Liu R., Xu G. (2015) The expression of CDK1 is associated with proliferation and can be a prognostic factor in epithelial ovarian cancer. Tumour Biol. 36, 4939–4948 10.1007/s13277-015-3141-8 25910705

[56] Costa-Cabral S., Brough R., Konde A., Aarts M., Campbell J., Marinari E. (2016) CDK1 is a synthetic lethal target for KRAS mutant tumours. PLoS ONE 11, e0149099 10.1371/journal.pone.0149099 26881434 PMC4755568

[57] Feng W., Cai D., Zhang B., Lou G. and Zou X. (2015) Combination of HDAC inhibitor TSA and silibinin induces cell cycle arrest and apoptosis by targeting survivin and cyclinB1/Cdk1 in pancreatic cancer cells. Biomed. Pharmacother. 74, 257–264 10.1016/j.biopha.2015.08.017 26349994

