# Supplementary material for: Overexpression of BUB1B, CCNA2, CDC20, and CDK1 in tumor tissues predicts poor survival in pancreatic ductal adenocarcinoma
Source: Biosci Rep. 2019 Feb 26;39(2):BSR20182306. doi: 10.1042/BSR20182306 (PMC6390130; doi:10.1042/BSR20182306)

Figure S1 Relative log expression (A) and Normalized unscaled standard errors (B) assessment of GSE46234

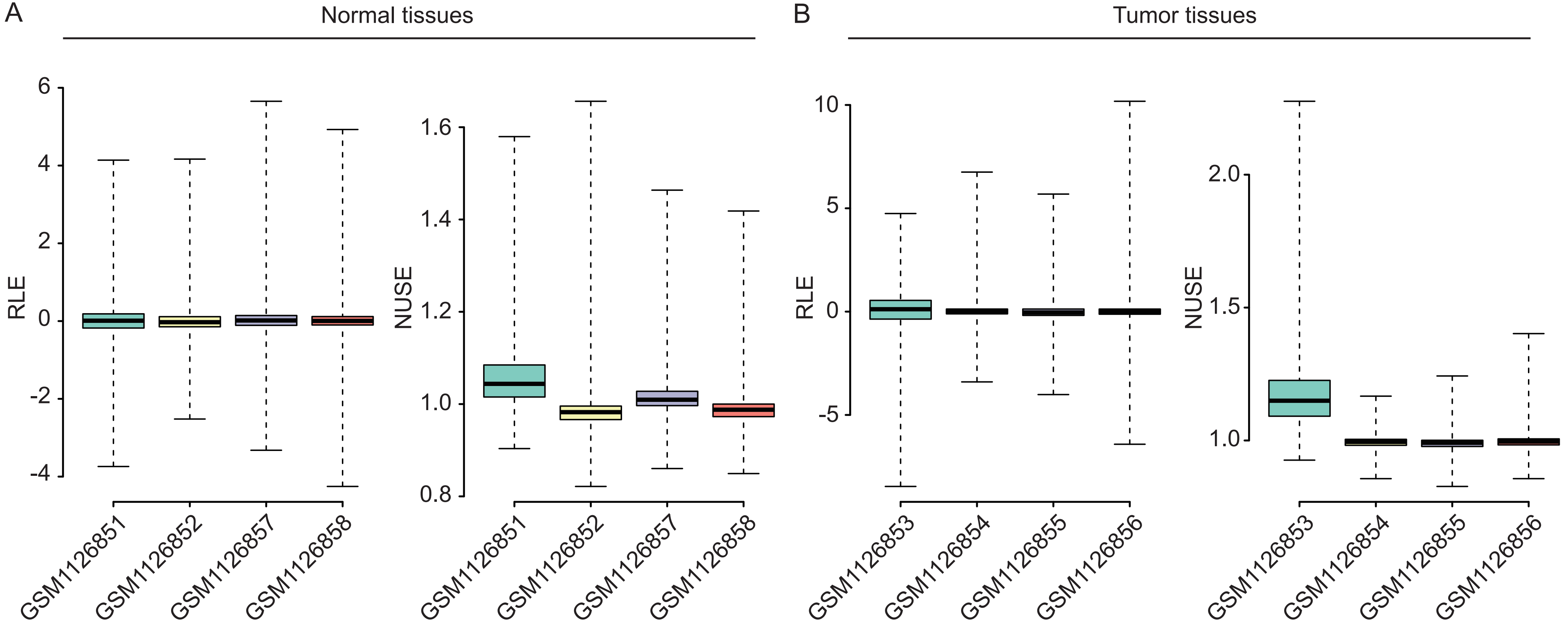

Figure S2 Relative log expression (A) and Normalized unscaled standard errors (B) assessment of GSE71989

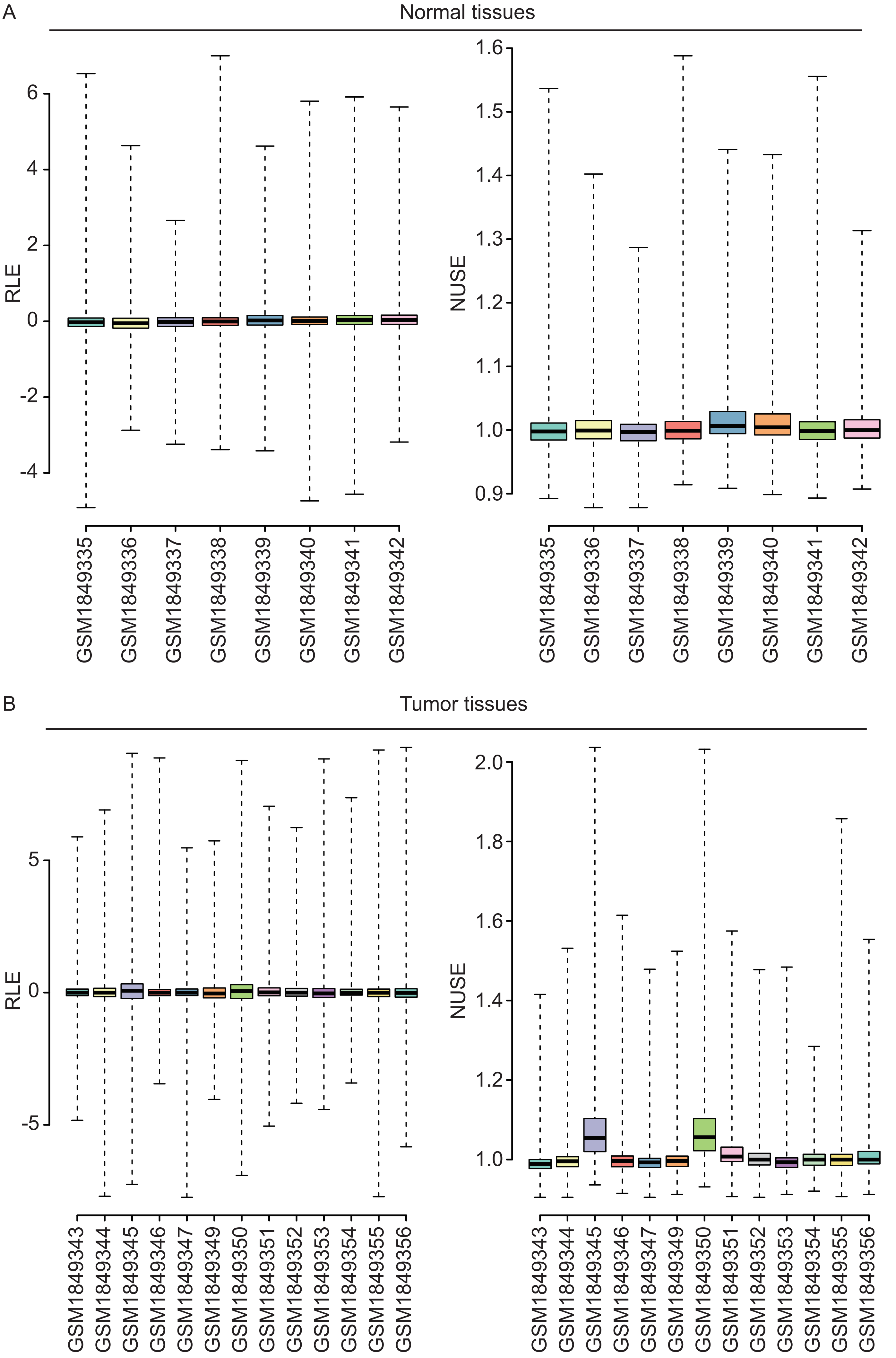

Figure S3 Relative log expression (A) and Normalized unscaled standard errors (B) assessment of GSE107610

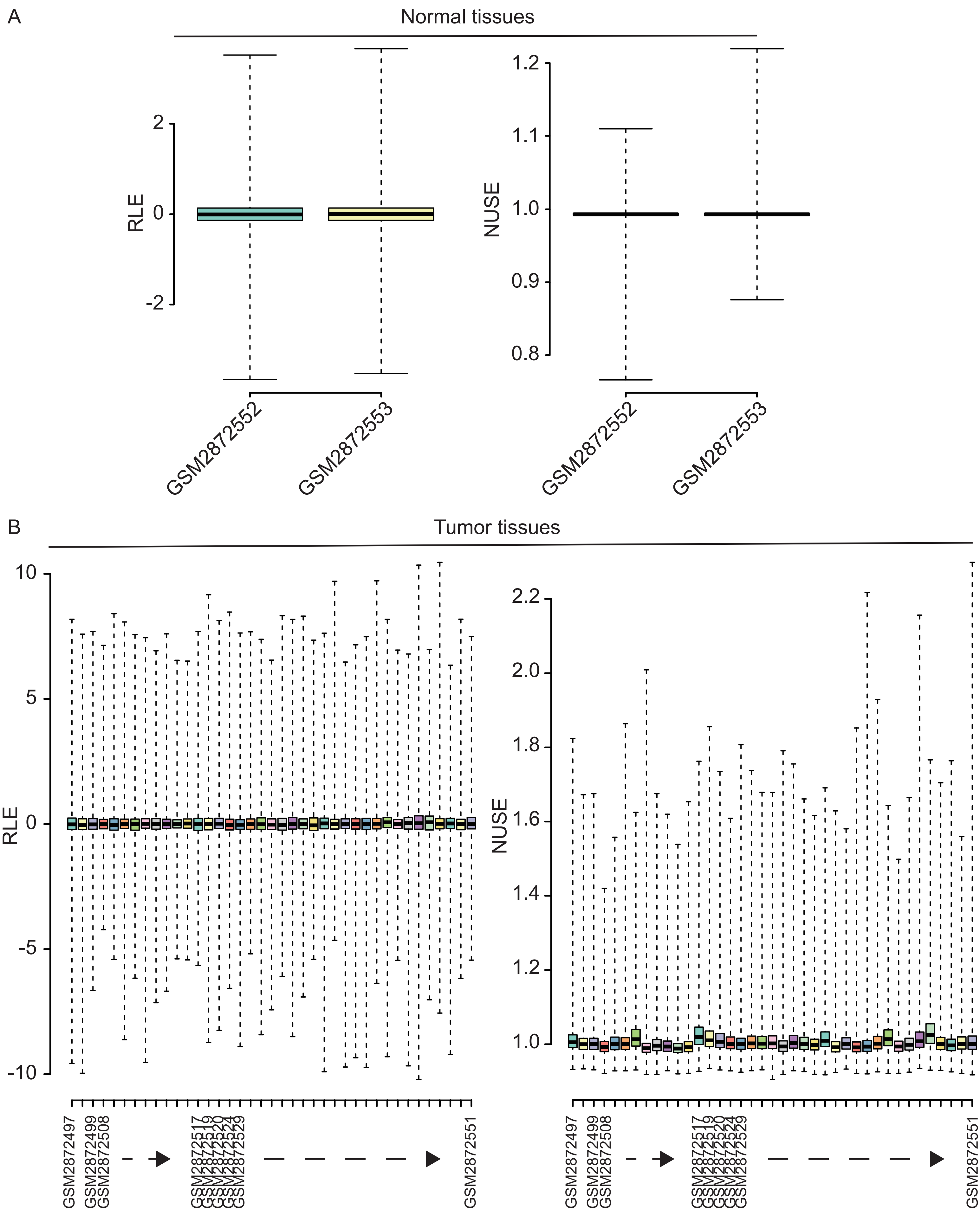

Supplement: Supplementary file 1 [file bsr-39-bsr20182306_Supp1.pdf]
